# Impact of polygenic schizophrenia-related risk and hippocampal volumes on the onset of psychosis

**DOI:** 10.1038/tp.2016.143

**Published:** 2016-08-09

**Authors:** F Harrisberger, R Smieskova, C Vogler, T Egli, A Schmidt, C Lenz, A E Simon, A Riecher-Rössler, A Papassotiropoulos, S Borgwardt

**Affiliations:** 1 Division of Neuropsychiatry and Brain Imaging, Department of Psychiatry (UPK), Psychiatric University Clinics Basel, University of Basel, Basel, Switzerland; 2 Psychiatric University Clinics, University of Basel, Basel, Switzerland; 3 Medical Image Analysis Centre, University Hospital Basel, Basel, Switzerland; 4 Division of Molecular Neuroscience, Department of Psychology, University of Basel, Basel, Switzerland; 5 King's College London, Department of Psychosis Studies, Institute of Psychiatry Psychology and Neuroscience, London, UK; 6 Specialized Early Psychosis Outpatient Service for Adolescents and Young Adults, Department of Psychiatry, Bruderholz, Switzerland; 7 Transfaculty Research Platform, University of Basel, Basel, Switzerland; 8 Department Biozentrum, Life Sciences Training Facility, University of Basel, Basel, Switzerland

## Abstract

Alterations in hippocampal volume are a known marker for first-episode psychosis (FEP) as well as for the clinical high-risk state. The Polygenic Schizophrenia-related Risk Score (PSRS), derived from a large case–control study, indicates the polygenic predisposition for schizophrenia in our clinical sample. A total of 65 at-risk mental state (ARMS) and FEP patients underwent structural magnetic resonance imaging. We used automatic segmentation of hippocampal volumes using the FSL-FIRST software and an odds-ratio-weighted PSRS based on the publicly available top single-nucleotide polymorphisms from the Psychiatric Genomics Consortium genome-wide association study (GWAS). We observed a negative association between the PSRS and hippocampal volumes (*β*=−0.42, *P*=0.01, 95% confidence interval (CI)=(−0.72 to −0.12)) across FEP and ARMS patients. Moreover, a higher PSRS was significantly associated with a higher probability of an individual being assigned to the FEP group relative to the ARMS group (*β*=0.64, *P*=0.03, 95% CI=(0.08–1.29)). These findings provide evidence that a subset of schizophrenia risk variants is negatively associated with hippocampal volumes, and higher values of this PSRS are significantly associated with FEP compared with the ARMS. This implies that FEP patients have a higher genetic risk for schizophrenia than the total cohort of ARMS patients. The identification of associations between genetic risk variants and structural brain alterations will increase our understanding of the neurobiology underlying the transition to psychosis.

## Introduction

Schizophrenia can be a severe mental disorder, affecting ~1% of the population.^1^ Although the pathophysiological mechanisms underlying schizophrenia are still poorly understood, it is known that genetic factors and combinations thereof (that is, single-nucleotide polymorphisms (SNP), copy-number variations or mutations) are involved in disease aetiology, as is indicated by the substantial heritability estimates for schizophrenia.^2^ Moreover, in combination with environmental trigger factors, it might lead to the transition to psychosis from the clinical high-risk state. Around 30% of clinical at-risk mental state (ARMS) individuals will make a transition to psychosis within the subsequent 2 years.^3, 4, 5^ Finding markers that further characterise these ARMS individuals is the main goal of psychiatric research, as early treatment of this group is thought to prevent or delay the onset of a first episode of psychosis.^6, 7^ Several markers besides clinical characteristics describe prodromal psychosis, for example, structural and functional brain alterations or cognitive functioning.^8, 9^ Even in the ARMS, neuroimaging observations revealed reductions in the grey matter of the medial temporal lobe, including the hippocampus,^10, 11, 12, 13, 14^ as well as neurofunctional aberrations within the hippocampus^15^ and deficits in verbal fluency and memory functioning.^16^ However, results are inconsistent in the differences in hippocampal volume between first-episode psychosis (FEP) patients and ARMS individuals, regardless of future transition to psychosis.^10, 11, 17^ Moreover, hippocampal volumes were shown to be highly heritable in twin studies of healthy individuals;^18, 19^ however, twin studies where one of the twins was affected by schizophrenia also revealed substantial modulation of hippocampal volumes by environmental factors.^20, 21, 22, 23^ In addition, moderate genetic heritability of the hippocampal volumes was shown in large extended families affected with schizophrenia.^24^

Although individual effects of SNPs on the genetic risk for schizophrenia were found to be small, it was estimated that 23% of variation in liability to schizophrenia is captured by SNPs with a substantial proportion of this variation attributed to common causal variants.^25, 26^ The largest genome-wide association study (GWAS), performed by the Psychiatric Genomic Consortium (PGC), identified 108 schizophrenia-associated loci,^27^ which explained up to 3.4% of the phenotypic variance in case–control studies. In general, the combination of GWAS-significant risk SNPs, the Polygenic Schizophrenia-related Risk Score (PSRS), describes the estimated cumulative genomic risk for schizophrenia.

Only a few studies have reported associations between a PSRS and brain volumes. All of these studies investigated the above-mentioned association in different cohorts of schizophrenia patients, their relatives and/or healthy controls.^28, 29, 30^ They found association of a PSRS with total brain volume,^28^ especially with white matter volume.^28, 29^ Unfortunately, these results could not be replicated in another independent sample.^30^ However, none of these studies investigated the association of a PSRS with brain volume in ARMS individuals and FEP patients. Moreover, a GWAS identified single SNPs linked to hippocampal volume in healthy controls;^18^ however, no study to date has investigated the association of a PSRS with volumetric differences in this region.

On the basis of findings supporting a role for hippocampal alterations in FEP and even in the ARMS,^10, 11, 12, 13, 14^ we aimed to explore the association between the PSRS, hippocampal volume and the onset of psychosis. The identification of associations between genetic risk variants and structural alterations will increase our understanding of the neurobiology underlying psychosis, as well as the transition to psychosis. Linking the PSRS to structural alterations in the brain will be helpful in elucidating the neurobiology underlying psychosis and may also increase our understanding of the factors contributing to the transition to psychosis in ARMS individuals. We hypothesised that a higher PSRS is associated with both smaller hippocampal volumes and the probability of being FEP.

## Materials and methods

### Participants and clinical assessment

Individuals included in this study were recruited via the early detection of psychosis research programme at the Psychiatry Outpatient Department, Psychiatric University Clinics Basel^5, 31^ and were either ARMS individuals or FEP patients. All individuals were assessed using the Basel Screening Instrument for Psychosis,^32^ the Brief Psychiatric Rating Scale (BPRS), the Scale for the Assessment of Negative Symptoms (SANS) and the Global Assessment of Functioning (GAF) at the time of the magnetic resonance imaging scan. We additionally obtained information on current and previous psychotropic medication, nicotine and illegal drug consumption using a semistructured interview adapted from the Early Psychosis Prevention and Intervention Centre Drug and Alcohol Assessment Schedule (eppic.org.au).

ARMS was defined in accordance with the criteria by Yung *et al.*^33^ and resulted in the inclusion of *N*=43 ARMS individuals in the study. Thus, inclusion required one or more of the following: (a) 'attenuated' psychotic symptoms, (b) brief limited intermittent psychotic symptoms or (c) a first- or second-degree relative with a psychotic disorder plus at least two indicators of a clinical change, according to the Basel Screening Instrument for Psychosis.^31, 34^ Inclusion because of criterion (a) required a change in the mental state at least several times a week and for more than 1 week (a score of 2 or 3 on the BPRS hallucination item or 3 or 4 on BPRS items for unusual thought content or suspiciousness). Inclusion because of (b) required BPRS scores of ⩾4 on the hallucination item or ⩾5 on the unusual thought content, suspiciousness or conceptual disorganisation items, with each symptom lasting less than 1 week before resolving spontaneously. None of the included subjects fulfilled criterion (c). All individuals were antipsychotic-naive at the time of scanning, whereas 18 of the ARMS individuals were receiving antidepressants.

The FEP patients (*N*=36) met the operational criteria according to Yung *et al.*,^33^ and they fulfilled criteria for acute psychotic disorder according to International Statistical Classification of Diseases, 10th Revision (ICD-10) or Diagnostic and Statistical Manual of Mental Disorders, Fifth Edition (DSM-5) but not for schizophrenia. Inclusion required scores of ⩾4 on the hallucination item or ⩾5 on the unusual thought content, suspiciousness or conceptual disorganisation items of the BPRS. The symptoms had to have occurred at least several times a week and persisted for more than 1 week. Fourteen of our FEP patients were antipsychotic-naive, three were antipsychotic-free and ten were receiving antipsychotic medication at the time of scanning (three quetiapine, three risperidone, two olanzapine, one clozapine and one aripiprazole). In the antipsychotic-free group, antipsychotic medication (two risperidone and one aripiprazole) has been stopped 4, 19 and 24 months previously. Antipsychotic dose was converted into chlorpromazine (CPZ) equivalents using the Supplementary Table Antipsychotic dose conversion by Ho *et al.*^35^ The mean CPZ equivalents (s.d.) were 227.39 (202.90). Of all FEP patients, three received only antidepressants and four were on a combined treatment with antidepressants and antipsychotics.

The following exclusion criteria were applied for both groups: history of previous psychotic disorder, psychotic symptomatology secondary to an ‘organic' disorder, psychotic symptoms associated with an affective psychosis or a borderline personality disorder, substance abuse according to ICD-10 research criteria, head trauma, neurological illness, serious medical or surgical illness, being younger than 18 years, inadequate knowledge of the German language and IQ less than 70 as measured by the Mehrfachwahl Wortschatz (Multiple Choice Vocabulary) Test Form B (MWT-B).

All participants provided written informed consent and received compensation for participating. The studies had permission from the ethics committee beider Basel (EKBB).

### Magnetic resonance imaging acquisition

All anatomical scans were performed on a 3 T magnetic resonance imaging scanner (Siemens Magnetom Verio, Siemens Healthcare, Erlangen, Germany) using a 12-channel phased-array radio frequency head coil. For structural images, a 3D T_1_-weighted magnetisation-prepared rapid gradient echo sequence was used with the following parameters: an inversion time of 1 000 ms, flip angle=8 degrees, repetition time (TR)=2 s, echo time (TE)=3.37 ms, field of view (FOV)=25.6 cm, acquisition matrix=256 × 256 × 176, resulting in 176 contiguous sagittal slices with 1 × 1 × 1 mm^3^ isotropic spatial resolution. All scans were screened for gross radiological abnormalities by an experienced neuroradiologist.

### Genotyping and imputation

DNA was extracted from whole-blood samples using the QIAamp DNA Blood Maxi kit according to the standard procedures (Qiagen, Chatsworth, CA, USA). DNA samples were further processed on the Affymetrix Genome-Wide Human SNP Array 6.0. in one centralised microarray facility as described in the Genome-Wide Human SNP Nsp/Sty 6.0. User Guide (Affymetrix, Santa Clara, CA, USA). Generation of SNP calls and array quality control (QC) were performed using the Affymetrix Genotyping Console Software 3.0 (Affymetrix). According to the manufacturer's recommendation, contrast QC was chosen as QC metric, using the default value of 0.4. All samples passing QC criteria were subsequently genotyped using the Birdseed (v2) algorithm, leading to a total of 921 523 genotyped SNPs per sample. Appropriate SNP QC filtering was applied in the PLINK 1.9 software,^36, 37^ where the gender check in PLINK led to the exclusion of three individuals.

Population stratification was assessed using principal component (PC) analysis implemented in the EIGENSTRAT software^38^ to detect genotypic outliers (with default parameters: >6 s.d.'s on any of the top 10 PCs in five iterations) and to correct for the potential population substructure by analysing all array-based pruned, autosomal SNPs. Eight individuals were identified as outliers and therefore were excluded from further analyses.

Before autosome-wide genotype imputation, haplotype estimation was performed using SHAPEITv2 software,^39^ allowing a per individual and a per SNP missing rate for observed markers of max. 5%. After pre-phasing, genotype imputation was performed using IMPUTE v2.3.0 software, which imputes missing genotypes using a multipopulation reference panel.^40, 41^ The integrated variant callset of 1092 individuals from the 1000 Genomes Project (release v3 in NCBI build 37/hg19 coordinates, March 2012) served as panel data (http://mathgen.stats.ox.ac.uk/impute/ALL_1000G_phase1integrated_v3_impute_macGT1.tgz). Only genotype calls exceeding a probability score of 90% were converted into genotype calls for statistical analysis using the PLINK 1.9 software.^42^

### PSRS calculation

PSRS were calculated, following the suggestions by Wray *et al.*,^43^ by taking linkage disequilibrium (LD)-pruned loci identified by the Schizophrenia Working Group of the PGC in a GWAS of 36 989 schizophrenia patients and 113 075 healthy controls^27^ (http://www.med. unc.edu/pgc/downloads). A total of 87 SNPs that could be mapped to one of the top SNPs of the 108 loci associated with schizophrenia and that survived QC were used to calculate the PSRS. (The following were included: 17 SNPs represented on the Affymetrix 6.0 Genotyping Array and 70 imputed SNPs (see Supplementary Table 1). The following were excluded: 7 SNPs that could not be imputed, 3 SNPs on allosome, 11 insertion/deletion variants and 20 variants in physically dependent genomic regions.). In summary, the number of risk alleles per person was weighted for each SNP by the logarithm of its odds ratio as reported in the PGC SZ data set^27^ and summed across SNPs^44^ using the PLINK 1.9 software.^36, 37^ The PSRS was then corrected for the first 20 genotypic PCs and the number of SNPs used to calculate the PSRS by using the z-transformed residuals of a linear regression.

### Image processing

Subcortical structures were segmented from T_1_-weighted magnetisation-prepared rapid gradient echo images with FMRIB's Integrated Registration and Segmentation Tool 5.0.4. (FSL-FIRST).^45^ Raw volumes for the left and right hippocampi were extracted and separately corrected for intracranial volume, age, gender, antidepressant intake as dichotomous variable and CPZ equivalents of antipsychotics by using the z-transformed residuals of a linear regression. After a separate outlier control for both hippocampal sides (mean±3.5 s.d.), which resulting in the exclusion of three individuals, the mean hippocampal volume was calculated.

### Statistical analysis

The R 3.0.2 software^46^ with the packages stats was used for statistical, group-related analysis. *Χ*^2^-tests or *t*-tests were used to test the distribution between diagnosis group and age, sex, handedness, years of education, IQ, BPRS, SANS, Global Assessment of Functioning, antipsychotics and antidepressants. Values are presented as mean±s.d. (see Table 1). In addition, associations between clinical symptoms and PSRS or hippocampal volumes were examined with linear regression analysis. The relationship between corrected PSRS (corrected for the first 20 genotypic PCs and the number of SNPs used to calculate the PSRS) and the corrected bilateral hippocampal volumes (corrected for intracranial volume, age, gender, antidepressant intake as dichotomous variable and CPZ equivalents of antipsychotic doses) was assessed in a linear regression model. We then fitted a logistic regression using the generalised linear model function in R with diagnosis status as binary dependent variable and the corrected bilateral hippocampal volumes and the corrected PSRS score as independent variables (both having similar variance between groups). Furthermore, mediation analysis^47, 48, 49^ was conducted to assess the driving factor of these associations using the R package mediation.^50^ The indirect effect was tested using the quasi-Bayesian Monte Carlo method based on normal approximation and the 95% confidence interval (CI) was obtained through 1000 simulations.

## Results

### Clinical and demographic characteristics

There were no significant differences among the investigated groups with respect to gender (*P*=0.83), handedness (*P*=0.11), years of education (*P*=0.96) MWT-B (*P*=0.74), SANS (*P*=0.27) and number of individuals treated with antidepressants (*P*=0.14). There were significant between-group differences in age (*P*=0.01), BPRS (*P*=0.001), Global Assessment of Functioning (*P*=0.009) and the number of patients treated with antipsychotics (*P*<0.001; Table 1). None of the clinical characteristics was associated with the PSRS or the hippocampal volumes at the time of MR scanning.

### Association between diagnosis, PSRS and hippocampal volume

**Figure 1:**
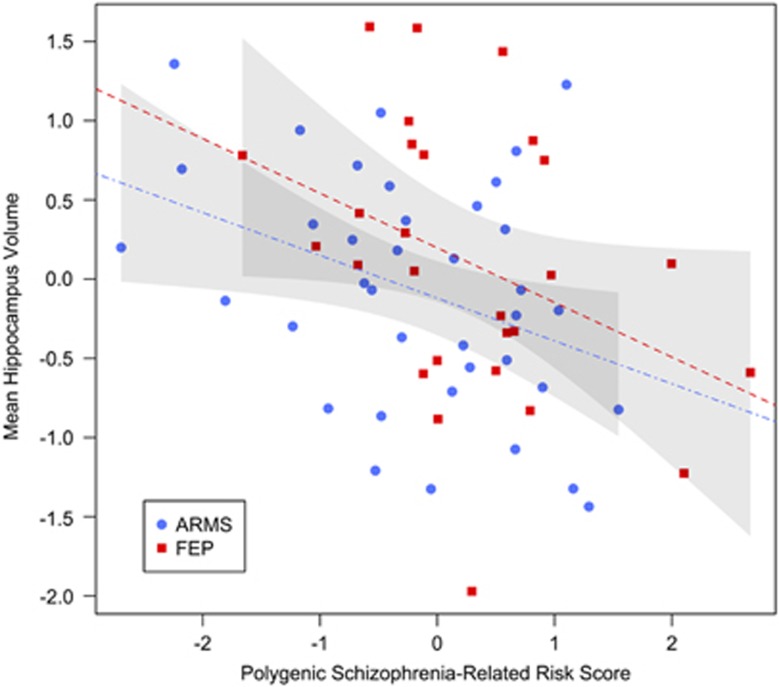
Linear regression analysis of PSRS and hippocampal volumes. Standardised residuals of the PSRS are adjusted for the first 20 genotypic PCs and the number of SNPs used to calculate the PSRS. Standardised residuals of the mean hippocampal volume are adjusted on each side separately for ICV, age, gender antidepressant intake and CPZ equivalents. Red dashed line, regression line with 95% confidence interval of FEP cohort; blue dot-dashed line, regression line with 95% confidence interval of ARMS cohort. ARMS, at-risk mental state; CPZ, chlorpromazine; FEP, first-episode psychosis; ICV, intracranial volume; PC, principal component; PSRS, Polygenic Schizophrenia-Related Risk Score; SNP, single-nucleotide polymorphism.

**Figure 2:**
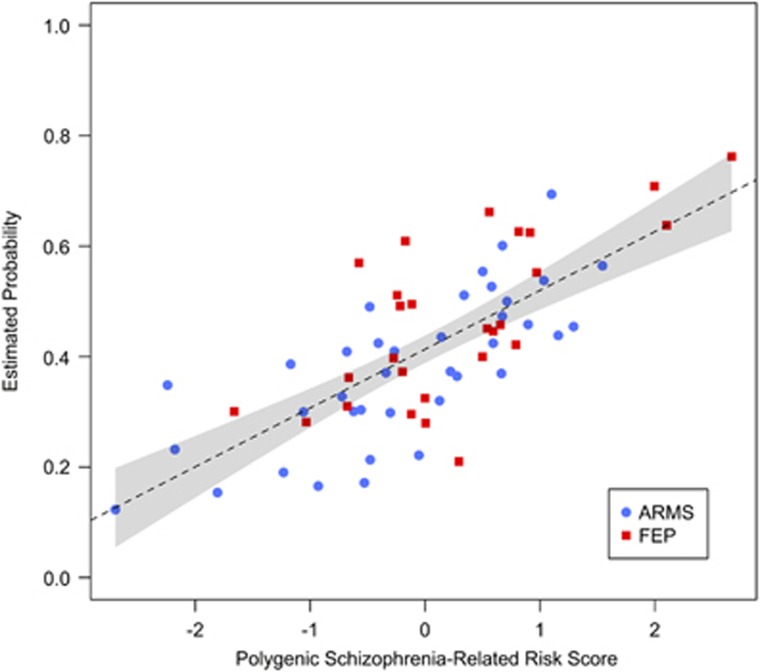
Plot of estimated probability for being FEP versus PSRS. The standardised residuals of the PSRS are adjusted for the first 20 genotypic PCs, and the number of SNPs used to calculate the PSRS are plotted against estimated probability of logistic regression. Black dashed line, regression line with 95% confidence interval of FEP and ARMS cohorts. ARMS, at-risk mental state; FEP, first-episode psychosis; PC, principal component; PSRS, Polygenic Schizophrenia-Related Risk Score; SNP, single-nucleotide polymorphism.

**Figure 3:**
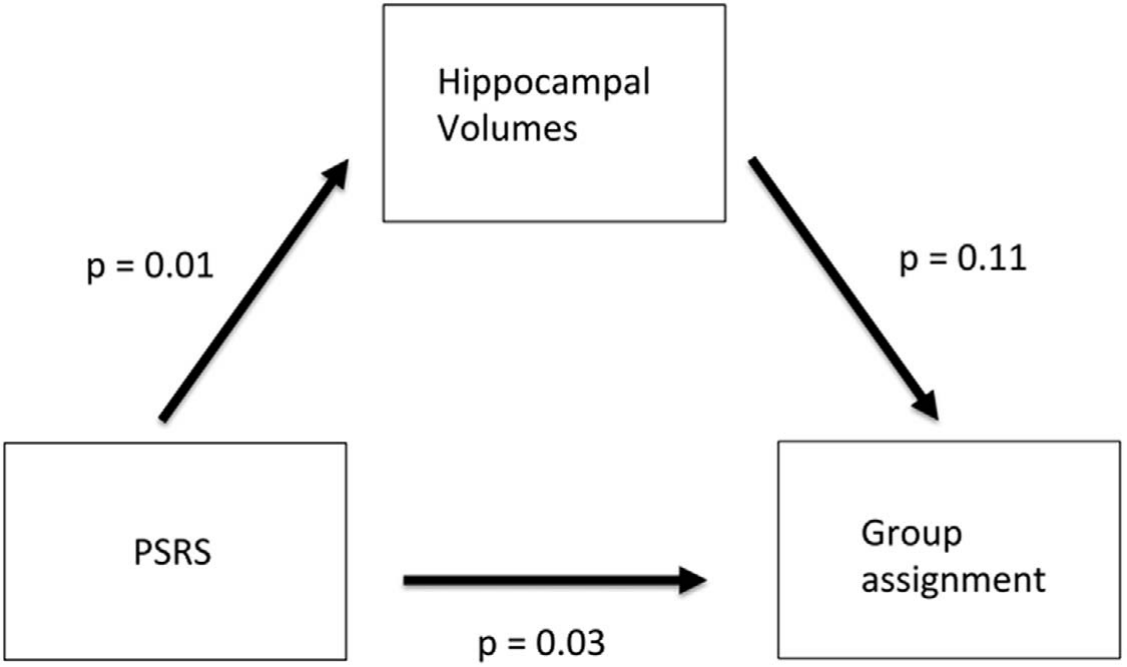
Mediation analysis scheme. Indirect effect of PSRS on group assignment through hippocampal volumes and direct effect of PSRS on group assignment. *P*-values are reported as estimate of significance. PSRS, Polygenic Schizophrenia-related Risk Score.

Linear regression analysis revealed a significant relationship between the PSRS and hippocampal volumes (*β*=−0.42, *P*=0.01, 95% CI=(−0.72 to −0.12), Table 2) in our total sample and the subgroup of ARMS individuals (*β*=−0.51, *P*=0.02, 95% CI=(−0.94 to −0.08), Figure 1, Table 2) and FEP patients separately (*β*=−0.41, *P*=0.05, 95% CI=(−0.83 to 0.01), Figure 1, Table 2). To further analyse this association in the total sample, we performed a logistic regression analysis. A significant main effect of the PSRS on the log odds of an individual being assigned to the FEP state was observed (*β*=0.64, *P*=0.03, 95% CI=(0.08–1.29), Table 2, Figure 2). In addition, neither a main effect of the hippocampal volumes (*β*=0.59, *P*=0.11, 95% CI=(−0.11 to 1.36), Table 2) nor an interaction effect of PSRS and hippocampal volumes (*β*=−0.14, *P*=0.70, 95% CI=(−0.88 to 0.60), Table 2) on the log odds was detected. Therefore, a higher PSRS score is associated with a higher likelihood that an individual would be assigned to the group of FEP individuals than to the group of ARMS individuals. Moreover, the mediation analysis indicated no mediating role of the hippocampal volumes between PSRS and group assignment (*β*=−0.03, *P*=0.09, 95% CI=(−0.09 to 0.006), Figure 3, Table 2). And, the direct effect of PSRS on group assignment when controlling for hippocampal volumes remained significant (*β*=0.14, *P*=0.03, 95% CI=(0.02–0.27), Figure 3, Table 2).

## Discussion

To our knowledge, this is the first study to analyse the association between a PSRS, hippocampal volumes and the onset of psychosis. We found a negative association between the hippocampal volumes and the PSRS across ARMS individuals and FEP patients, derived from the top hits within genome-wide significant loci identified by the large GWAS analysis from the Psychiatric Genomics Consortium.^27^ Moreover, a higher PSRS was significantly associated with a higher probability of being assigned to the FEP group than to the ARMS group.

We demonstrate that reduced hippocampal volumes were associated with higher PSRS in the total sample of ARMS individuals and FEP patients as well as for each group separately. This association might suggest that schizophrenia-related SNPs are directly linked to smaller hippocampi. However, such a direct link cannot be inferred from our results because other factors such as stressful life events^51^ or neuropsychiatric medication^52, 53^ have been shown to modulate the volumes of the hippocampus. It should be further noted that volumetric alterations in the hippocampus have been linked to psychotic symptoms and cognitive deficits of schizophrenia,^54^ a core function of the hippocampus, and ARMS individuals already show some deficits in verbal fluency and memory functioning.^5, 16^

We also observed that a higher PSRS was associated with a higher likelihood of an individual being assigned to the FEP group than to the ARMS group. This finding might reflect the fact that only ~30% of ARMS individuals are correctly predicted to develop psychosis^4, 5^ and thus might not have a high PSRS. Moreover, the hippocampal volumes were not identified as mediator between PSRS and group assignment. Therefore, further studies should analyse whether the PSRS could be used to further characterise those ARMS individuals who will develop psychosis and whether ARMS individuals with a higher PSRS are more likely to develop psychosis. Owing to the limited number of ARMS individuals with later transition to psychosis, we could not investigate whether this PSRS might be a vulnerability trait for transition. Nevertheless, we observed that four of our six ARMS individuals who (until now) have developed psychosis had a PSRS above the median of the total sample. Therefore, further longitudinal studies should examine whether a combination of clinical, genetic, environmental, neuroimaging and neurocognitive markers can improve the prediction rate for transition to psychosis.

The absence of a significant association between hippocampal volumes and being in either the ARMS or FEP groups supports several findings of similar volumes.^10, 11, 17^ Furthermore, it has been reported that the volumes of the hippocampus were negatively associated with negative symptoms in ARMS individuals and schizophrenia patients^55, 56, 57, 58^ and that the hippocampal–prefrontal pathway was linked to negative symptoms and cognitive deficits in schizophrenia.^59^ Therefore, it might be speculated that the similar levels of negative symptoms in FEP patients and ARMS individuals might partially underlie the absence of volumetric hippocampal differences. However, future functional and structural connectivity studies should further examine the hippocampus and the inter-related cortical and subcortical regions, including the dorsolateral prefrontal cortex to assess possible impairments in neuronal networks in schizophrenia. Moreover, it was demonstrated that a PRS was associated with negative symptoms and not positive symptoms in a large sample of adolescence from the general population.^60^ Therefore, it might be especially important to focus on the combined analysis of clinical, genetic and neuroimaging data.

### Limitations

There are some limitations to bear in mind concerning the results of this study. First, the sample size is relatively small. However, the groups are homogeneous with regard to genetic background and clinical characteristics related to disease status and prognosis.^61^ This makes confounding effects of disease duration or antipsychotic medication unlikely. In addition, polygenic risk scores derived from large GWAS generate robust estimators,^62^ which can be used in small samples. Second, the PSRS explains only a small amount of variance in liability to schizophrenia and cannot be considered as a classifier between ARMS individuals and FEP patients. Thus, prediction of actual transition to psychosis is not possible; however, this aspect will be further investigated when we have obtained enough follow-up data. Third, as the aim of the present study was to include patients with a first psychotic episode independent of the underlying diagnosis according to ICD/DSM classification systems, no conclusions can be drawn regarding non-affective versus affective psychoses specifically.

## Conclusion

In summary, this is the first study to evaluate a negative association between a PSRS and hippocampal volumes in ARMS individuals and FEP patients. Our findings suggest that the combination of a subset of schizophrenia risk variants is related to hippocampal volume and that higher values of this genome-wide significant PSRS (but not hippocampal volume or the interaction effect) are associated to FEP status than to the ARMS. These findings imply that FEP patients have a higher genetic risk for schizophrenia than the total cohort of ARMS individuals, and encourage further studies on the use of PSRS as an additional marker in the prediction of psychosis from the prodromal state.

## Figures and Tables

**Table 1:** Demographics and clinical characteristics

*Characteristics*	*ARMS (*n*=38)*	*FEP (*n*=27)*	*Statistics*	P*-value*
Gender M/F (% M)	26/12 (32%)	20/7 (26%)	*χ*^2^=0.05	0.83
Mean age in years (s.d.)	23.83 (4.31)	28.33 (7.91)	*t*=−2.68	0.01
Handedness r/l (% l)	35/3 (8%)	20/7 (26%)	*χ*^2^=2.68	0.11
Years of education (s.d.)	13.72 (2.59)	13.76 (3.15)	*t*=−0.05	0.96
MWT-B (s.d.)	110.73 (13.85)	109.23 (17.88)	*t*=0.33	0.74
BPRS (s.d.)	37.16 (7.28)	50.33 (15.49)	*t*=−3.90	0.001
SANS (s.d.)	19.55 (15.31)	24.14 (15.15)	*t*=−1.13	0.27
GAF (s.d.)	70.11 (12.35)	59.59 (17.07)	*t*=2.73	0.009
AP no/yes (% y)	38/0 (0%)	17/10 (37%)	*χ*^2^=13.91	<0.001
AD no/yes (% y)	20/18 (47%)	20/7 (26%)	*χ*^2^=2.23	0.14

Abbreviations: AD, antidepressants; AP, antipsychotics; ARMS, at-risk mental state; BPRS, Brief Psychiatric Rating Scale; F, female; FEP, first-episode psychosis; GAF, Global Assessment of Functioning; l, left; M, male; MWT-B, Mehrfachwahl Wortschatz Test (Multiple Choice Vocabulary) Form B; r, right; SANS, Scale for the Assessment of Negative Symptoms.

**Table 2:** Results of linear regression, logistic regression and mediation analyses

*Variable*	*Coefficients*	*s.e.*	Z-*value*	P*-value*	*95% CI lower*	*95% CI upper*
*Linear regression: ARMS and FEP*						
Intercept	0.02	0.12	0.15	0.88	−0.22	0.26
Hippocampal volumes	−0.42	0.15	−2.83	0.01	−0.72	−0.12
*R*^2^=0.11; comparison with null model: *χ*^2^=7.75, *P*=0.01						
						
*Linear regression: ARMS only*						
Intercept	−0.20	0.16	−1.29	0.21	−0.52	0.12
Hippocampal volumes	−0.51	0.21	−2.39	0.02	−0.94	−0.08
*R*^2^=0.14; comparison with null model: *χ*^2^=5.60, *P*=0.02						
						
*Linear regression: FEP only*						
Intercept	0.32	0.17	1.82	0.08	−0.04	0.68
Hippocampal volumes	−0.41	0.20	−2.03	0.05	−0.83	0.01
*R*^2^=0.14; comparison with null model: *χ*^2^=4.11, *P*=0.04						
						
*Logistic regression: ARMS and FEP*						
Intercept	−0.43	0.29	−1.48	0.14	−1.01	0.13
PSRS	0.64	0.30	2.11	0.03	0.08	1.29
Hippocampal volumes	0.59	0.37	1.60	0.11	−0.11	1.36
PSRS x hippocampal volumes	−0.14	0.37	−0.39	0.70	−0.88	0.60
Nagelkerk'se-*R*^2^=0.1; *c*-statistic: 64.4% comparison with null model: *χ*^2^=5.88, *P*=0.12						
						
*Mediation analysis*						
ACME	−0.03			0.09	−0.09	0.006
ADE	0.14			0.03	0.02	0.27
Total effect	0.12			0.07	−0.01	0.25

Abbreviations: ARMS, at-risk mental state; ACME, average causal mediation effect; ADE, average direct effect; CI, confidence interval; FEP, first-episode psychosis; PSRS, Polygenic Schizophrenia-related Risk Score.

## Supplementary Material

Supplementary Informations
