# Supplementary material for: Impact of polygenic schizophrenia-related risk and hippocampal volumes on the onset of psychosis
Source: Transl Psychiatry. 2016 Aug 9;6(8):e868–. doi: 10.1038/tp.2016.143 (PMC5022088; doi:10.1038/tp.2016.143)
Supplement: Supplementary Informations [file tp2016143x1.doc]

**Supplementary Information:**

**Supplementary Table 1.** Summary data of included loci

| **SNP** | **Allele** | **Chromosome** | **Position** | **Directly genotyped** |
| --- | --- | --- | --- | --- |
| rs4648845 | TC | 1 | 2,372,401-2,402,501 |  |
| rs1498232 | TC | 1 | 30,412,551-30,437,271 |  |
| rs11210892 | AG | 1 | 44,029,384-44,128,084 | x |
| rs12129573 | AC | 1 | 73,766,426-73,991,366 |  |
| rs1702294 | TC | 1 | 98,374,984-98,559,084 |  |
| rs140505938 | TC | 1 | 149,998,890-150,242,490 |  |
| rs6670165 | TC | 1 | 177,247,821-177,300,821 |  |
| rs7523273 | AG | 1 | 207,912,183-208,024,083 | x |
| rs77149735 | AG | 1 | 243,555,105-243,555,105 |  |
| rs11682175 | TC | 2 | 57,943,593-58,065,893 |  |
| rs3768644 | AG | 2 | 72,357,335-72,368,185 |  |
| rs2909457 | AG | 2 | 162,798,555-162,910,255 |  |
| rs11693094 | TC | 2 | 185,601,420-185,785,420 |  |
| rs59979824 | AC | 2 | 193,848,340-194,028,340 |  |
| rs6434928 | AG | 2 | 198,148,577-198,835,577 |  |
| rs6704641 | AG | 2 | 200,161,422-200,309,252 | x |
| rs11685299 | AC | 2 | 225,334,096-225,467,796 |  |
| rs6704768 | AG | 2 | 233,559,301-233,753,501 | x |
| rs17194490 | TG | 3 | 2,532,786-2,561,686 |  |
| rs4330281 | TC | 3 | 17,221,366-17,888,266 |  |
| rs75968099 | TC | 3 | 36,843,183-36,945,783 |  |
| rs2535627 | TC | 3 | 52,541,105-52,903,405 |  |
| rs832187 | TC | 3 | 63,792,650-64,004,050 |  |
| rs7432375 | AG | 3 | 135,807,405-136,615,405 |  |
| rs215411 | AT | 4 | 23,366,403-23,443,403 | x |
| rs35518360 | AT | 4 | 103,146,888-103,198,090 |  |
| rs10520163 | TC | 4 | 170,357,552-170,646,052 | x |
| rs1106568 | AG | 4 | 176,851,001-176,875,801 | x |
| rs1501357 | TC | 5 | 45,291,475-45,393,775 |  |
| rs4391122 | AG | 5 | 60,499,143-60,843,543 |  |
| rs16867576 | AG | 5 | 88,581,331-88,854,331 |  |
| rs4388249 | TC | 5 | 109,030,036-109,209,066 | x |
| rs3849046 | TC | 5 | 137,838,092-137,948,092 | x |
| rs111294930 | AG | 5 | 152,097,521-152,323,121 |  |
| rs11740474 | AT | 5 | 153,671,057-153,688,217 |  |
| rs1339227 | TC | 6 | 73,132,701-73,171,901 |  |
| rs117074560 | TC | 6 | 96,459,651-96,459,651 |  |
| rs12704290 | AG | 7 | 86,403,226-86,459,326 |  |
| rs6466055 | AC | 7 | 104,598,064-105,063,064 |  |
| rs211829 | TC | 7 | 110,034,393-110,106,693 |  |
| rs13240464 | TC | 7 | 110,843,815-111,205,915 |  |
| rs7801375 | AG | 7 | 131,539,263-131,567,263 |  |
| rs3735025 | TC | 7 | 137,039,644-137,085,244 |  |
| rs10503253 | AC | 8 | 4,177,794-4,192,544 |  |
| rs73229090 | AC | 8 | 27,412,627-27,453,627 |  |
| rs6984242 | AG | 8 | 60,475,469-60,954,469 |  |
| rs7819570 | TG | 8 | 89,340,626-89,753,626 |  |
| rs36068923 | AG | 8 | 111,460,061-111,630,761 |  |
| rs4129585 | AC | 8 | 143,309,503-143,330,533 | x |
| rs11139497 | AT | 9 | 84,630,941-84,813,641 |  |
| rs7893279 | TG | 10 | 18,681,005-18,770,105 | x |
| rs11191419 | AT | 10 | 104,585,135-104,956,335 |  |
| rs11027857 | AG | 11 | 24,367,320-24,412,990 | x |
| rs9420 | AG | 11 | 57,386,294-57,682,294 |  |
| rs12421382 | TC | 11 | 109,285,471-109,610,071 |  |
| rs2514218 | TC | 11 | 113,317,794-113,423,994 | x |
| rs77502336 | CG | 11 | 123,394,636-123,395,986 |  |
| rs55661361 | AG | 11 | 124,610,007-124,620,147 |  |
| rs10791097 | TG | 11 | 130,714,610-130,749,330 | x |
| rs75059851 | AG | 11 | 133,808,069-133,852,969 |  |
| rs4240748 | CG | 12 | 92,243,186-92,258,286 | x |
| rs2068012 | TC | 14 | 30,189,985-30,190,316 |  |
| rs2332700 | CG | 14 | 72,417,326-72,450,526 |  |
| rs2693698 | AG | 14 | 99,707,919-99,719,219 |  |
| rs12887734 | TG | 14 | 103,996,234-104,184,834 |  |
| rs56205728 | AG | 15 | 40,566,759-40,602,237 |  |
| rs12903146 | AG | 15 | 61,831,663-61,909,663 |  |
| rs12148337 | TC | 15 | 70,573,672-70,628,872 |  |
| rs8042374 | AG | 15 | 78,803,032-78,926,732 |  |
| rs950169 | TC | 15 | 84,661,161-85,153,461 |  |
| rs4702 | AG | 15 | 91,416,560-91,429,040 |  |
| rs9922678 | AG | 16 | 9,875,519-9,970,219 |  |
| rs7405404 | TC | 16 | 13,728,459-13,761,359 |  |
| rs12691307 | AG | 16 | 29,924,377-30,144,877 |  |
| rs12325245 | AT | 16 | 58,669,293-58,682,833 |  |
| rs8044995 | AG | 16 | 67,709,340-68,311,340 |  |
| rs4523957 | TG | 17 | 2,095,899-2,220,799 |  |
| rs8082590 | AG | 17 | 17,722,402-18,030,202 |  |
| rs9636107 | AG | 18 | 53,195,247-53,200,117 | x |
| rs72934570 | TC | 18 | 53,453,389-53,585,689 |  |
| rs2905426 | TG | 19 | 19,374,022-19,658,022 |  |
| rs2053079 | AG | 19 | 30,981,643-31,039,023 |  |
| rs56873913 | TG | 19 | 50,067,499-50,135,399 |  |
| rs6065094 | AG | 20 | 37,361,494-37,485,994 | x |
| rs7267348 | TC | 20 | 48,114,136-48,131,649 |  |
| rs9607782 | AT | 22 | 41,408,556-41,675,156 |  |
| rs6002655 | TC | 22 | 42,375,814-42,689,414 |  |
